# Concurrent Bouveret's Syndrome and Gallstone Ileus: A Rare Case of Dual-Site Obstruction in Gallstone Disease

**DOI:** 10.7759/cureus.90990

**Published:** 2025-08-25

**Authors:** Abhilash Mesh, Ahmed Aly, Mohammed Elmorsy, Gayan Nanayakkara

**Affiliations:** 1 Department of General Surgery, Withybush Hospital, Haverfordwest, GBR

**Keywords:** bouveret’s syndrome, gallstone, gastrointestinal obstruction, gi radiology, surgery

## Abstract

Bouveret's syndrome is a rare cause of gastric outlet obstruction, resulting from gallstone migration into the duodenum via a biliary-enteric fistula. Even rarer is its coexistence with distal gallstone ileus. We report the case of an elderly woman presenting with gastric outlet obstruction who was found to have dual-site obstruction caused by two impacted gallstones. Despite multidisciplinary management involving imaging, endoscopy, and surgery, her postoperative course was complicated by sepsis and multiorgan failure, leading to death. This case underscores the severe morbidity and mortality associated with advanced gallstone disease in elderly patients with comorbidities and highlights the challenges in timely diagnosis and treatment.

## Introduction

Gallstone ileus is a rare mechanical complication of cholelithiasis that occurs when one or more gallstones erode through the gallbladder wall into the gastrointestinal tract via a cholecystoenteric fistula. It accounts for approximately 1-4% of all mechanical small bowel obstructions, rising to 25% in patients over 65 years of age [1].

Bouveret's syndrome, first described by Léon Bouveret in 1896, is a rare variant in which a large gallstone migrates through a biliary-enteric fistula to obstruct the gastric outlet or proximal duodenum [2]. This condition represents less than 3% of all cases of gallstone ileus [3]. Patients typically present with nonspecific upper gastrointestinal symptoms, including nausea, vomiting, epigastric pain, and features of gastric outlet obstruction.

Even more unusual is the simultaneous presence of Bouveret's syndrome and distal gallstone ileus, representing multiple stones lodging at different sites. This phenomenon suggests the passage of multiple calculi through a single fistulous tract, which is rarely reported and poses additional challenges in diagnosis and management.

## Case presentation

An 84-year-old female presented to the emergency department with lower abdominal pain, persistent vomiting, and clinical features of sepsis. On arrival, she was haemodynamically unstable, with hypotension (BP 82/50 mmHg) and tachycardia. Her past medical history included hypertension, type 2 diabetes mellitus, stage 3 chronic kidney disease, and hyperlipidaemia.

Laboratory investigations demonstrated evidence of systemic inflammation, haemoconcentration, and acute renal failure. Findings included marked leukocytosis with neutrophilia, elevated CRP (C-reactive protein), severe renal impairment with reduced eGFR (estimated glomerular filtration rate), and electrolyte disturbance (hyponatraemia). Liver function tests were largely normal apart from a mild ALP (alkaline phosphatase) rise. These results were consistent with sepsis, dehydration, and acute-on-chronic kidney injury (Table 1).

**Table 1:** Blood results of the patient on admission Abbreviations for laboratory parameters: WBC = White Blood Cell, Hb = Haemoglobin, PLT = Platelets, RBC = Red Blood Cell, Hct = Haematocrit, MCV = Mean Cell Volume, MCH = Mean Cell Haemoglobin, RDW = Red Cell Distribution Width, CRP = C-reactive Protein, ALP = Alkaline Phosphatase, ALT = Alanine Transaminase, eGFR = Estimated Glomerular Filtration Rate Abbreviations for units of measurement: g/L = grams per litre, pg = picograms, fL = femtolitres, U/L = units per litre, mg/L = milligrams per litre, mmol/L = millimoles per litre, µmol/L = micromoles per litre, ×10⁹/L = billions per litre, ×10¹²/L = trillions per litre, mL/min/1.73 m² = millilitres per minute per 1.73 square metres of body surface area

Parameter	Result	Units	Reference Range
White Blood Cell (WBC) Count	14.8	×10⁹/L	4.0–11.0
Haemoglobin (Hb)	171	g/L	115–165
Platelet (PLT) Count	308	×10⁹/L	150–400
Red Blood Cell (RBC) Count	6.25	×10¹²/L	3.80–5.50
Haematocrit (Hct)	0.52	L/L	0.37–0.47
Mean Cell Volume (MCV)	83	fL	80–100
Mean Cell Haemoglobin (MCH)	27.2	pg	27.0–33.0
Red Cell Distribution Width (RDW)	16.2	%	11.0–14.8
Neutrophil Count	13	×10⁹/L	1.7–7.5
Lymphocyte Count	0.9	×10⁹/L	1.0–4.5
Monocyte Count	0	×10⁹/L	0.2–0.8
Eosinophil Count	0	×10⁹/L	0.0–0.4
Basophil Count	0	×10⁹/L	0.0–0.1
Amylase	115	U/L	<100
C-reactive Protein (CRP)	191	mg/L	<5
Calcium (Total)	2.51	mmol/L	2.20–2.60
Calcium (Adjusted)	2.65	mmol/L	2.20–2.60
Albumin	35	g/L	35–50
Total Protein	66	g/L	60–80
Globulin	31	g/L	35–50
Bilirubin	16	µmol/L	<21
Alkaline Phosphatase (ALP)	163	U/L	30–130
Alanine Transaminase (ALT)	20	U/L	<33
Urea	27.8	mmol/L	2.5–7.8
Sodium	126	mmol/L	133–146
Potassium	5.1	mmol/L	3.5–5.3
Creatinine	306	µmol/L	46–92
Estimated Glomerular Filtration Rate (eGFR)	13	ml/min/1.73 m²	>60

**Figure 1:**
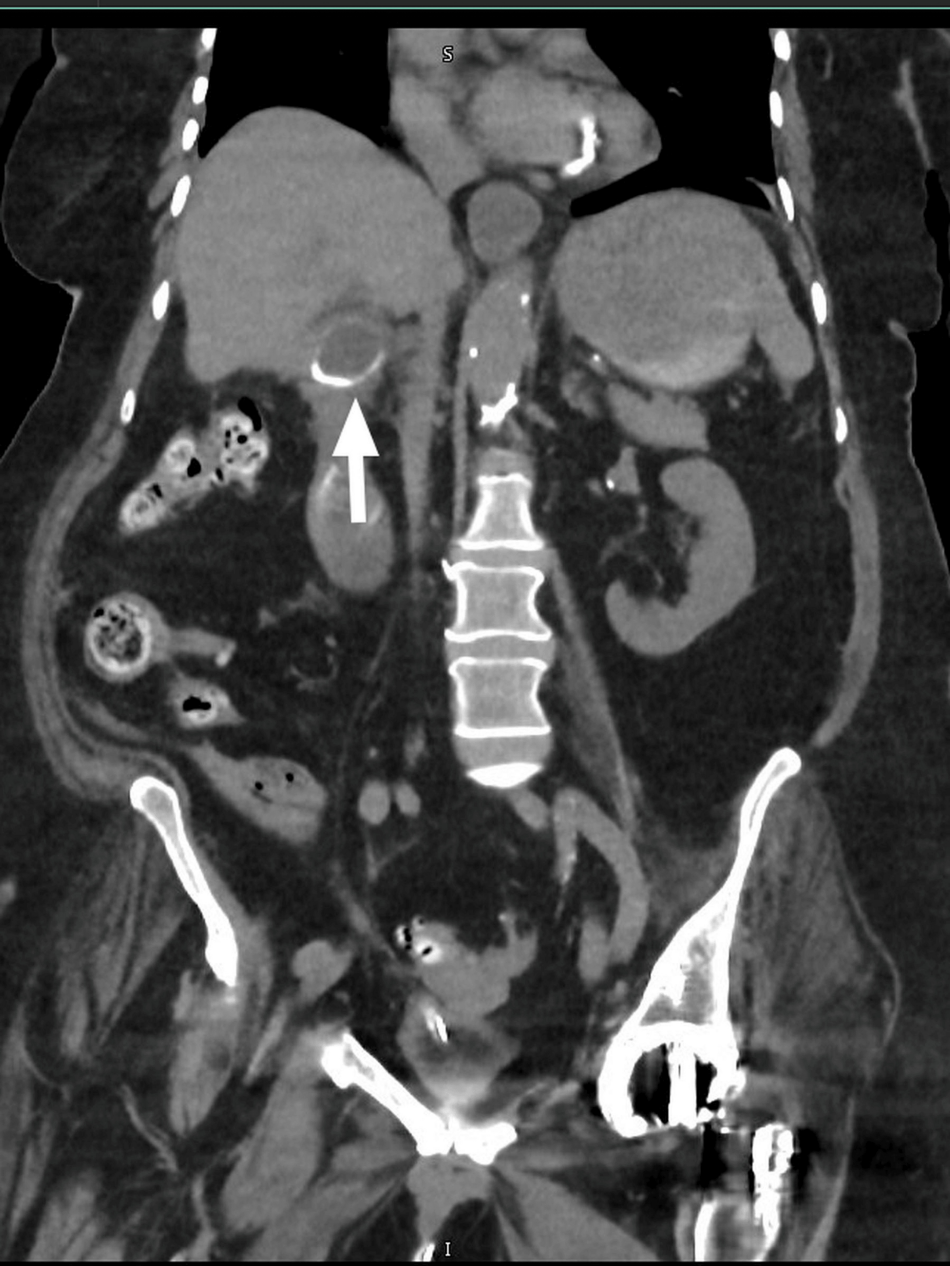
Coronal image Coronal CT image of the abdomen showing a large 3.3 cm calcified gallstone (white arrow) impacted at the gallbladder neck. The gallbladder appears collapsed, and there is associated pneumobilia, supporting the presence of a cholecystoenteric fistula.

**Figure 2:**
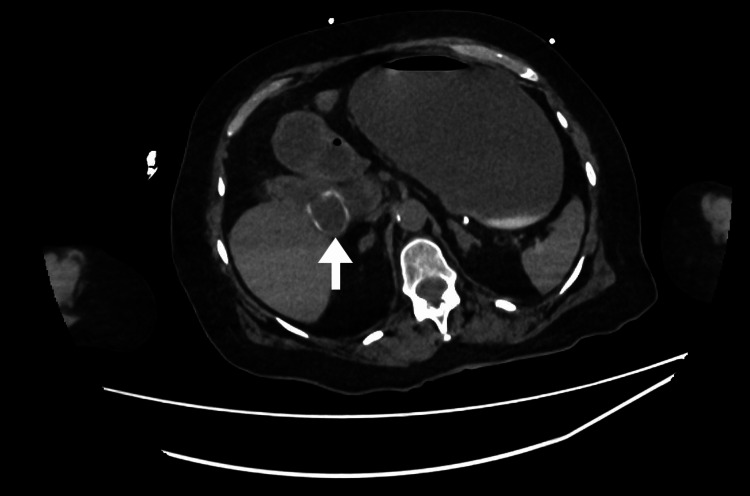
Axial image A second stone, measuring 2.6 cm, was located in the third portion of the duodenum (D3) (white arrow).

**Figure 3:**
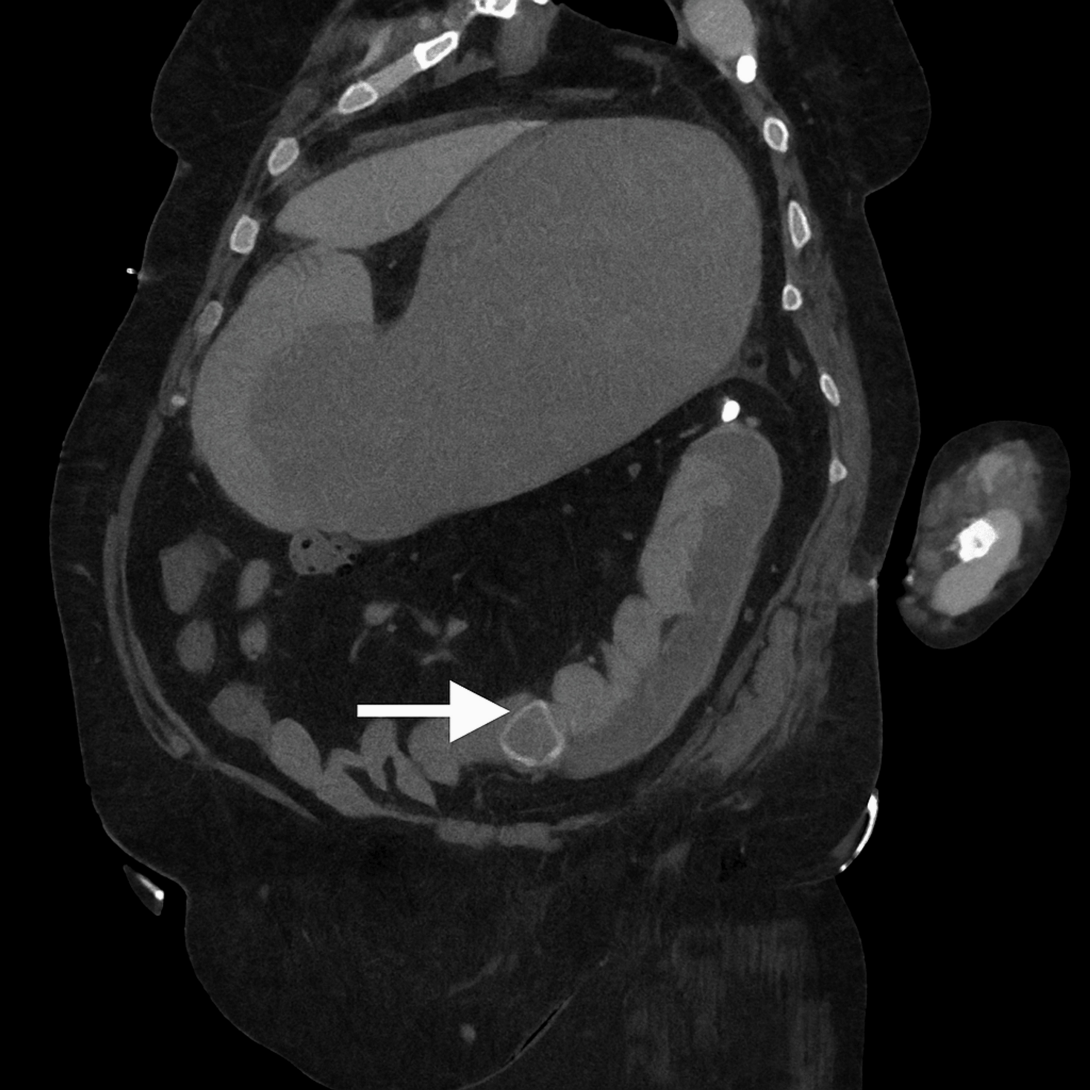
Coronal image Coronal CT image of the abdomen and pelvis demonstrating a 3.2 cm ectopic gallstone (white arrow) impacted in a loop of distal ileum, resulting in proximal small bowel dilatation consistent with gallstone ileus and mechanical obstruction.

Cross-sectional imaging demonstrated classical features of gallstone ileus, including pneumobilia and a suspected choledochoduodenal fistula. The gallbladder was contracted and collapsed, containing a 3.3 cm calcified gallstone impacted at the neck (Figure 1). A second stone, measuring 2.6 cm, was located in the third portion of the duodenum (D3) (Figure 2). A third stone, measuring 3.2 cm, was visualised within a distal ileal loop (Figure 3), associated with proximal small bowel dilatation, consistent with mechanical small bowel obstruction. These findings are consistent with Rigler's triad, comprising pneumobilia, ectopic gallstone, and intestinal obstruction, and support the diagnosis of gallstone ileus secondary to chronic cholelithiasis with fistulisation.

A midline laparotomy was performed, revealing an obstructing gallstone within the distal ileum. The affected segment was opened, and the stone was retrieved via enterotomy, which was subsequently closed primarily. On palpation, a large second gallstone was identified impacted in the second/third part of the duodenum.

An intraoperative oesophagogastroduodenoscopy (OGD) was attempted to facilitate endoscopic removal. Despite balloon catheter manipulation and repeated combined attempts by two surgeons using simultaneous palpation and endoscopic guidance, the stone could not be mobilised.

Due to persistent impaction, a 3 cm longitudinal gastrotomy was fashioned just proximal to the pylorus to provide access to the duodenum. Multiple retrieval techniques were required, including balloon catheters, a Foley catheter, and Desjardin's forceps, before the 2.6 cm stone was successfully extracted intact from the duodenum. The gastrotomy was closed with a linear stapler reinforced with interrupted PDS sutures, and haemostasis was secured.

Given the patient's intraoperative frailty and comorbid status, no attempt was made to repair the cholecystoenteric fistula or perform a cholecystectomy.

Following surgery, the patient was transferred to the high-dependency unit (HDU) for close monitoring and advanced postoperative care. Initially, she remained haemodynamically stable, with adequate urine output and no immediate surgical complications noted. She was managed with intravenous fluids, broad-spectrum antibiotics, analgesia, and serial monitoring of inflammatory markers and renal function. Early enteral nutrition was withheld, and she remained nil by mouth with nasogastric decompression.

Over the next 48 hours, however, her condition began to deteriorate. She developed worsening tachycardia, increasing oxygen requirements, and oliguria, prompting escalation to critical care input. Investigations revealed worsening acute kidney injury, rising inflammatory markers, and new-onset metabolic acidosis. A diagnosis of progressive multi-organ dysfunction was made, likely secondary to systemic inflammatory response syndrome (SIRS) and sepsis, despite no clear evidence of an anastomotic leak or intra-abdominal collection on repeat imaging.

By postoperative day five, the patient required vasopressor support for hypotension and was commenced on renal replacement therapy for worsening renal function. She was intubated and mechanically ventilated due to hypoxic respiratory failure. Blood cultures grew *Escherichia coli*, and antibiotics were escalated based on the sensitivity results.

Despite maximal supportive measures, including multi-organ support, continuous renal replacement therapy (CRRT), and critical care intervention, her condition continued to decline. Repeat imaging showed no surgically correctable cause of deterioration, and discussions were held with the family regarding prognosis.

On postoperative day 10, the patient developed refractory hypotension and cardiac arrest. Resuscitation was attempted but was unsuccessful. She was declared deceased following discussion with the intensive care team and her next of kin.

## Discussion

Bouveret's syndrome remains a diagnostic challenge due to its rarity and nonspecific presentation. It may mimic more common causes of gastric outlet obstruction, such as peptic ulcer disease, gastric malignancy, or pancreatitis, often resulting in delayed recognition [1,2]. This is particularly true in elderly patients, where vague symptoms and multiple comorbidities may obscure the clinical picture.

Computed tomography (CT) is the imaging modality of choice and plays a critical role in diagnosis. It allows for the identification of Rigler's triad, which includes pneumobilia, ectopic gallstone, and intestinal obstruction, present in only 30-50% of cases [3-5]. CT is especially valuable for visualising multiple stones and fistula formation, as well as assessing the level and severity of obstruction [5,6].

Endoscopic management is often the first-line intervention for Bouveret's syndrome, particularly in frail or high-risk surgical candidates. Modalities such as mechanical lithotripsy, electrohydraulic lithotripsy, and laser lithotripsy have been employed with varying degrees of success (10-40%) [7-9]. However, large or impacted stones often necessitate surgical intervention.

When gallstone ileus is concurrently present, surgical management becomes imperative. Surgical options include enterolithotomy alone or combined procedures with cholecystectomy and fistula closure. In elderly or unstable patients, a limited approach focused on stone removal alone is often preferred to minimise operative time and risk, with delayed fistula repair considered on a case-by-case basis.

The coexistence of Bouveret's syndrome and distal gallstone ileus is extremely rare and further complicates both diagnosis and management. A multidisciplinary approach involving surgeons, radiologists, and gastroenterologists is essential for timely intervention and improved patient outcomes.

Critical reflection on the fatal outcome

In this case, despite the technically successful retrieval of both obstructing gallstones, the patient unfortunately succumbed in the postoperative period. Several factors likely contributed. First, the patient's advanced age, frailty, and comorbidities reduced physiological reserve. Second, the prolonged operative course, with repeated unsuccessful endoscopic and surgical retrieval attempts, may have exacerbated operative stress, anaesthetic exposure, and fluid shifts. Third, the need for both enterotomy and gastrotomy increased the risk of postoperative complications such as leaks, sepsis, and delayed recovery.

Reported mortality for gallstone ileus ranges from 12% to 27% and is higher still when Bouveret's syndrome coexists, reflecting both diagnostic delay and the complexity of management. In this context, the patient's outcome was consistent with the high-risk nature of this dual pathology.

The main lesson from this experience is the importance of tailoring intervention to patient physiology rather than aiming for technical completeness. While definitive surgery, including cholecystectomy and fistula closure, may be advocated in fit patients, a limited procedure may be the only realistic and appropriate option in unstable or frail patients. Early recognition, preoperative optimisation, minimising operative time, and anticipatory postoperative critical care support are crucial to improving survival.

## Conclusions

This case illustrates a rare and complex presentation of Bouveret's syndrome with concurrent distal gallstone ileus, underscoring the diagnostic and therapeutic challenges posed by multiple obstructing gallstones. Timely diagnosis requires a high index of suspicion and appropriate imaging, particularly in elderly patients presenting with nonspecific gastrointestinal symptoms. Although endoscopic techniques are often considered the first line of treatment in high-risk patients due to their minimally invasive nature, success rates remain low for large or impacted stones. In such situations, and especially in the presence of dual-site obstruction, surgical intervention remains the cornerstone of management. The extent of surgery should be tailored to the patient's physiological reserve and intraoperative findings; in elderly or frail patients, a limited approach focused on stone extraction, without cholecystectomy or fistula repair, may offer the safest balance between efficacy and risk.

Ultimately, optimal outcomes in this rare clinical scenario depend on early multidisciplinary involvement, careful risk-benefit assessment, and individualised treatment strategies. This case contributes to the limited body of literature on dual-site gallstone obstruction and reinforces the importance of awareness, early recognition, and judicious operative planning in managing complex complications of gallstone disease.

## References

[1] Parvataneni S, Khara HS, Diehl DL (2020). Bouveret syndrome masquerading as a gastric mass-unmasked with endoscopic luminal laser lithotripsy: a case report. World J Clin Cases.

[2] Navarro-Del Río E, Hernández-Zúñiga JF (2020). Bouveret's syndrome: a rarest complication of cholelithiasis. A case report and literature review. Cir Cir.

[3] Varre JS, Wu JL, Hopmann P, Ruiz O, Reddy R (2021). Endoscopic and surgical management of Bouveret's syndrome complicated by gallstone ileus. J Surg Case Rep.

[4] Bruni SG, Pickup M, Thorpe D (2017). Bouveret's syndrome—a rare form of gallstone ileus causing death: appearance on post-mortem CT and MRI. BJR Case Rep.

[5] Ferhatoğlu MF, Kartal A (2020). Bouveret's syndrome: a case-based review, clinical presentation, diagnostics and treatment approaches. Sisli Etfal Hastan Tip Bul.

[6] Caldwell KM, Lee SJ, Leggett PL, Bajwa KS, Mehta SS, Shah SK (2018). Bouveret syndrome: current management strategies. Clin Exp Gastroenterol.

[7] Chang L, Chang M, Chang HM, Chang AI, Chang F (2018). Clinical and radiological diagnosis of gallstone ileus: a mini review. Emerg Radiol.

[8] Dumonceau JM, Devière J (2016). Novel treatment options for Bouveret's syndrome: a comprehensive review of 61 cases of successful endoscopic treatment. Expert Rev Gastroenterol Hepatol.

[9] Rey Chaves CE, Villamil CJ, Ruiz S, Galvis V, Conde D, Sabogal Olarte JC (2022). Cholecystogastric fistula in Bouveret syndrome: case report and literature review. Int J Surg Case Rep.

